# Supplementary material for: Patterns of Intron Gain and Loss in Fungi
Source: PLoS Biol. 2004 Nov 30;2(12):e422. doi: 10.1371/journal.pbio.0020422 (PMC532390; doi:10.1371/journal.pbio.0020422)
Supplement: Table S1 — Also available at http://genes.mit.edu/NielsenEtAl/. (4.3 MB ZIP). [file pbio.0020422.st001.zip › NielsenEtAl/html/820.html]

AN3751.1.NCU00161.1.MG05695.1.FG05442.1


```
 CLUSTAL W (1.82) Multiple Sequence Alignments - Introns Inserted


Sequence 1: NCU00161.1	970 aa
Sequence 2: MG05695.1	833 aa
Sequence 3: FG05442.1	796 aa
Sequence 4: AN3751.1	883 aa
Alignment Length: 999 aa
Number Identitical Residues: 181 aa
Alignment Score (without introns) 10387


MG05695.1 	--MEPAMASGPVHTD-----------PGQAFQQPPLHTLGS------DQSGSRT------
NCU00161.1	MSMEPGTSGAPLEAEAAPVTVNGGTAPTHTPTQPQTSTLPTSTACPLSPSGPSTSASAVP
FG05442.1 	MAINPSTEPLPRIQS---------------------------------------------
AN3751.1  	MCPSPRTDADANAIAS----------PNGRQSAYAGGELSPSDSEDAGLGGSHYSSGGIG
          	 . .*     .    :          .             .: :   . ...  ::..  

MG05695.1 	---------------------------DTSEEEDDGVFSEGEAAQSLFQPAISPIATRLA
NCU00161.1	PKLDVPDPQVLSSTHSQPHEPSTTAAQNSDDNEDDLIYDDQDGAQSFIQPAISARAARLT
FG05442.1 	---------------------------TDVQEDEDSIICNQDEAEAILQPALSEAARHLT
AN3751.1  	--------------------------AAGAGIADLDDADDADEADDVIGVSMGYDQTRLK
          	                                 :     : : *: .:  ::.    :* 

MG05695.1 	WPGTPAAEKAAAMW--DFMDGPTDNIADSLSSNPSAGPNHGGVFVARPETPANMLNATLN
NCU00161.1	SPNTPAAEKAAAM---DYLPSLFDFMDDEGRAQSSGKTSGNPSPPMSPRKPQ--RRGHFR
FG05442.1 	SQNTPAIEKALALD--KSRAGFFGVPTDRVGDTSPDLSSSPETSPQVPQFPAS-AKSPVP
AN3751.1  	SRTTPAEEKTRIIERNGGYSGDEGLFAADITEEPESLADDGDQDKDENWRAPAGKNGTAE
          	   *** **:  :  .    .  .         .   ..           .    ..   

MG05695.1 	GASSVAVPHKPASVPTIAPQNRPQMPESGAGKLSNSGTPGFSSRK-RASTVGGDALKKLS
NCU00161.1	QSSSSRLPDLPTPWRAEPKQMIVGQP-HGAKTSSMFGVFGNETRSSRAASAGENALKKLS
FG05442.1 	TSPEPPQYALPSPWQARPKP-PVTKGHTSRPSGSILGNALAPARN-RSKSAGQEALRRLQ
AN3751.1  	TPVAKAQNLRTGFTRSILKGTLPLRPRAWSGD-SHTGSRSGLKKFFPSLHLRASSLSVSR
          	 .        .    :                 *  *      :   :     .:*    

MG05695.1 	RAIPSISMPSNFLPSLPTPSFFSSS--SHKRDTSLSPTRNGTCTKSGQVTTDHSFEESSL
NCU00161.1	KALPSISIP--------TPSFFSSSSSSHKESTSTWPSVP-HLLKSSTIAGIRSNRAQTG
FG05442.1 	RALPSLSPPTHLLPS-MSNSFFSSSQDKSSANHSTASSYSSPATR-QHSPRIPSNALGIA
AN3751.1  	YRSRSWSSR--------LNLDQEKGGDVSAAPQSPQSPQPPNPSSTASNGTLVTGTAPVE
          	    * *               ... .      *  ..       :       :      

MG05695.1 	AKEESIAIRYPHGPKPLRPLGRPGQAYTLRRSTSMESTLYNPMTRVSSYGDDERFLDVRK
NCU00161.1	PPLNTPGGATAQGPVRSSSL-RVSRTPTLRHSTSDDSLLYQSLTRVSSLGDDERFANVRE
FG05442.1 	QPLSQQTRRPASNLDELNTPSPPVRPKALRRVTSDDTRLYHSLSRTSSLGDDERFQDVRE
AN3751.1  	DPIGDLSPLQAPSARTRGKHSFVGSNPTLRRSSSDQSLYLRASSTASSLEHRPQYEHIHS
          	          . .       .      :**: :* ::   .. : .**  .  :: .::.

MG05695.1 	MTNSRFKAIMDSFDR--------PSFKMPQLPS-------------------MTSPFKSG
NCU00161.1	QVNTRIKAIIDSFE---------PSFKLPQLPKSTSFARLVAISRGKLTEAGITSPLKKV
FG05442.1 	MVNIRFMALKDSLPDV-------PNFKMPSLPK-------------------LYSQARKS
AN3751.1  	QTNSRFKAIKDTLQDSSSRLLSMPTLHLQDLRSDWGYKQFLSDAHHR---RTETNQTDDA
          	 .* *: *: *::   ::   : *.::: .* .. .     : :          .   . 

MG05695.1 	SFTESSIGS--VPRSPGKP-RNSTISP-KDGIAILDAVLESLTGDVVVLGGYRGSILRSA
NCU00161.1	VPSASDFHS--TDGTRSAP-GPSSHRPSRETVHPLDAILETLTGDIVVMGGYRGSILRSA
FG05442.1 	TISLGTSHTPDTPLNHSQV-PKSLDIDSQDGSAVLDRVLESLTGDLVIMGGYRGSVLRSA
AN3751.1  	LISTTSPPEANVHSPSAIPRAKSTRKSAAATYPVLFEAMSELTGDVVVMGGYRGSILRSA
          	  :      ...    .     *    :      *   :. ****:*::******:****

MG05695.1 	SSPHQQLWVPVKVGLNMRKVNLEVGLDPQDEETMEEHIIPSSMLQNIGPIDISRRLLRRM
NCU00161.1	EPPYRQLWVPVKVGLNIRKVNMEVGLEPEDEENMEKYIFASGMLQNIGPIDISRKLFRKL
FG05442.1 	EPPYHQVWAPVKLGLNMRKVNLEVGLEDEDEERMEETIKPDGMLKHIGPVDVSRKFIKKL
AN3751.1  	KPPHRQLWVPMKVGLNLRKVDLEVGLNPEDEERMEETVIPDGVLSHVGPVDICRRLLKRL
          	..*::*:*.*:*:***:***::****: :*** **: : ...:*.::**:*:.*::::::

MG05695.1 	RESRNAKSGTLRVHEFAYDWRLSPARSSARLLKFLEGLESN---VRDKPSGALVVAHSLG
NCU00161.1	RECENSVNGKLRVHDYGYDWRLSPHRLSKKLNEFIEKLPSNQPHVPPEQRGVWVIAHSLG
FG05442.1 	RSCDNAKSGKLRIWDYGYDWRLSPHLLSRKLQEYLQKLPSNQPGTPAGSRGALVISHSLG
AN3751.1  	QKCENAVRGELRVHNYGYDWRLSPHLLSRRLIKYLEGLPCNSPDIPPHKRGAYVIAHSLG
          	:.. *:  * **: ::.*******   * :* :::: * .*..       *. *::****

MG05695.1 	GLITRHAVNQRPNLFSGVVYAGVPQRAINIIG~PLRNGDAVLLNQKLLTAQVNFSFRTSF
NCU00161.1	GLITRHAVNQRPELYSGIVYAGVPNRCINILG2PLRNGDAVLFNETVLKAQSNFSFRSSF
FG05442.1 	GIITRHAVNQTPDLFSGVLYCGTPQRCVNILN~PLRHGDVVLLNEKLLTASVNFSMRTSF
AN3751.1  	GLITRHAVNQRPELFAGVLYAGVPQHCVNILG~PLRHGDDVLLSSRVLTAQVNFTFRTSF
          	*:******** *:*::*::*.*.*::.:**:. ***:** **:.. :*.*. **::*:**

MG05695.1 	VFLPEDGECFVSKDGTHEFKFDFYDPENWVKYRLCPAVAPPLKPALPVSKLDSALYSLRE
NCU00161.1	IFLPEDGFCFVSKDNKEEYPVDFYNVDDWIKYRLSPCAGGPALPPLNPSKSTGALGSLLS
FG05442.1 	VFLPEDGFCFVDKNTGEEYPIDFYDPYEWIRWHLSPCMQ-PALPPHNRPQSS----SFSS
AN3751.1  	ALLPEEGHCFINKRTKEEYRLNFFDPAVWDEFRLSPCINRPLPAPGLSSINLPLRKRFST
          	 :***:* **:.*   .*: .:*::   * .::*.*.   *  ..   .        :  

MG05695.1 	MST---TISTFDLRGKS-------------------------GSDDRGRKASILANGGVL
NCU00161.1	LSDSFPNLSSLPFRGRSNSGKNRTSDDGDDNNNNNNSSSSSSSSNNKHITASFSPSNTVF
FG05442.1 	FLP-----NSLRARAES-----------------------------RSEKGPPSPTAVAQ
AN3751.1  	MLSNSSTDDSIGAEDSTSD--------------------QSPTPSGHSTSNSVSAAAAAA
          	:  . .. .::  .  :..                    .:. ...:  . .  .   . 

MG05695.1 	GPPPLPTED-SGPHQTNGDDSRTSSPGSSQGQQEDQHYSRAYRYLARTLAETKQFRAETQ
NCU00161.1	KDRTLAPQMGSSSTATDTPETNPQQPAAPPINGTPSSHSQAIRYLERTLRETKQFRSELA
FG05442.1 	DHNPMAPQLNGSGAKVEEQKTS------------DLERKRYLDYLGRTLAATRKFRSELA
AN3751.1  	ASKIKPPKPKGEILPGPTTDPRGSSTTGPSTQPVTIPAAAARDYLARTLSEVLAFKRETL
          	     ..:  .        ..  ... ..  .           ** ***  .  *: *  

MG05695.1 	HRPELSDSNAYPPLAVIYGKDTPTVHTVRVSSLDAIASPDETYNDLVFRSGDGVVLAREA
NCU00161.1	HNPSHQHRNTYPPLAVIYAKDIPTVYAARVTCRDAIACTD-AYDDLMFASGDGVVLARES
FG05442.1 	HSPAHQETNAYPPHAVLYSKSIPTVYAAQVTSREGICCSD-AYDDLLFRPGDGVVLAKEA
AN3751.1  	HREDHQSSNRYPPFAVLYSKSVPTVYGARVNSRESIKYAD-AYDDLAFAAGDGVCLASAA
          	*    .  * *** **:*.*. ***: .:*.. :.*  .* :*:** * .**** **  :

MG05695.1 	MLPPGYDVVSGGRISTEKGHISILGDFVGVARAIEAVLRGRGKGIGKG------------
NCU00161.1	MLPEGYELVKDGRVCTDRGHVSMLGDLAAVGRALEAVVRGRRKGIGRGGVEEGEAEAVEA
FG05442.1 	MLPDGYSIVRGGRVCTERGHITMLGDIPAMGKALEALVRGRRKGIGMG------------
AN3751.1  	MLPTGYRVIRGGLVKSDRGHIGLLGDLEGVGACLKALVKGRKEGVGIG------------
          	*** ** :: .* : :::**: :***: .:. .::*:::** :*:* *            

MG05695.1 	-----VGAMDS-----------------------------
NCU00161.1	EKAEAVKALGGEGEGEGNSVGNNGVELQRTEQGTAVAERD
FG05442.1 	-----VGGDEAK----------------------------
AN3751.1  	-------AMNSS----------------------------
          	       .  ..
```
